# Prevalence and Predisposing Factors of Non-infectious Cardiac Implantable Electronic Device Lead Masses as Incidental Finding During Transoesophageal Echocardiography: A Retrospective Cohort Study

**DOI:** 10.3389/fcvm.2022.879505

**Published:** 2022-06-14

**Authors:** Tanja Kuecken, Ruta Jasaityte, Cara Bülow, Jessica Gross, Anja Haase-Fielitz, Michael Neuss, Christian Butter

**Affiliations:** Department of Cardiology, Heart Centre Brandenburg Bernau, Brandenburg Medical School (MHB) Theodor Fontane, Faculty of Health Sciences Brandenburg, Bernau, Germany

**Keywords:** incidental lead masses, cardiac implantable electronic device, transoesophageal echocardiography, CIED infection, anticoagulation

## Abstract

**Objectives:**

In this study, we assessed the prevalence and predisposing factors of non-infectious CIED lead masses as incidental finding during transoesophageal echocardiography (TOE).

**Methods:**

In a retrospective single centre study, we analysed TOE examinations performed for indications other than infectious endocarditis in 141 patients with CIED. Patients with non-suspicious leads and those with incidental non-infectious lead masses were compared with respect to clinical characteristics, anticoagulation, indication for TOE, and CIED lead characteristics. The odds ratios for non-infectious CIED lead masses were calculated.

**Results:**

Non-infectious CIED lead masses were detected in 39 (27.6%) of the 141 patients. They were more often identified on ICD and CRT-D leads compared to pacemaker and CRT-P leads [OR 2.77 (95% CI 1.29–5.95), *p* = 0.008]. The lifespan of the CIEDs from the first implantation to the index TOE did not differ between both groups. Incidental CIED lead masses were more prevalent in patients who received their device for primary prevention of sudden cardiac death (43.2%) and for resynchronisation (63.6%) but were less prevalent in patients with oral anticoagulation [OR.33 (95% CI.003–1.003), *p* = 0.048].

**Conclusion:**

Incidental non-infectious CIED lead masses were frequently found in TOE, with highest prevalence in ICD and CRT-D devices implanted for patients with dilated cardiomyopathy. Patients with therapeutic anticoagulation had significantly lower prevalence of CIED lead masses than those without.

## Introduction

In the last decade, the broadening spectrum of indications for cardiac implantable electronic devices (CIEDs) led to continuously rising numbers of device implantations (1–3). Concomitantly, infectious CIED-related endocarditis is becoming more prevalent, with reported incidence ranging from 0.6 to 3.45% and with an in-hospital or 30-day mortality rate as high as 5–8% when treated (4–6). The EHRA consensus document on CIED infections proposed a diagnostic framework based on the modified Duke criteria and ESC 2015 guidelines, where masses on intracardiac leads in echocardiography account for one of the major criteria (4). Subsequently, CIED removal and lead extraction are suggested in the presence of such echocardiographic findings when in combination with clinical signs of CIED infection (4, 5).

As such, incidentally detected oscillating masses on CIED leads in patients without apparent signs of systemic or device infection or thromboembolism present a diagnostic dilemma with serious consequences for further treatment. The reported prevalence of these findings is around 5–10%, with most of the masses not being related to endocarditis (7, 8). However, given the increasing number and longevity of the implanted CIEDs and dramatically increased spatial and temporal resolutions of echocardiographic 2D and 3D images, the number might be expected to be higher. Besides that, little is known about the incidence and patient- and CIED-associated predisposing factors of uninfected lead masses. Such information would be important for prevention and guidance in the decision-making of further diagnostic measures and treatment.

Thus, in this study, we aimed to define the current prevalence and predisposing factors of incidental non-infectious lead masses.

## Materials and Methods

### Patients

In this single-centre, retrospective cohort study, we reviewed consecutive transoesophageal echocardiographic (TOE) studies performed in the period from 1 January 2020 to 31 December 2021. From these, the TOE examinations performed on patients with CIEDs were selected. Patients with suspected infectious endocarditis or CIED infection were not considered for the study. If there was more than one transoesophageal study performed in the revised period for the same patient, only the first one was included in the study.

In case a CIED lead mass was detected, the presence or absence of device infection was identified from patient medical record. This decision was taken by the clinician board and was based on blood parameters of infection, positive blood cultures, Duke criteria, and local signs of device infection. Only patients with no signs of possible CIED infection were included in the final analysis.

**FIGURE 1:**
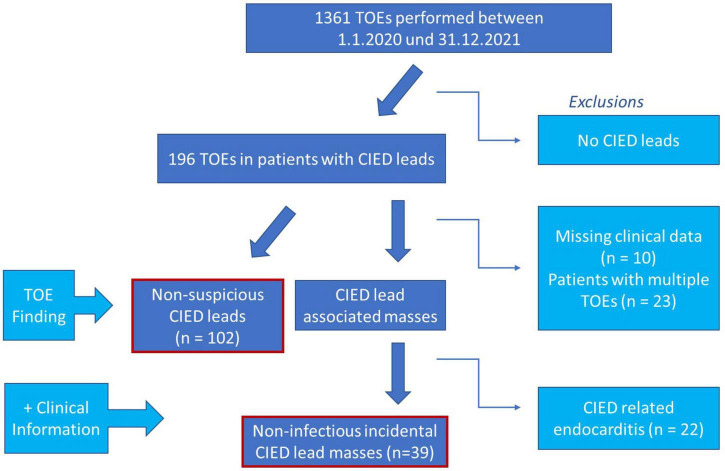
Graphic illustration of study design. CIED, cardiac implantable electronic device; TOE, transoesophageal echocardiography.

Patient flow is shown in Figure 1.

### Transoesophageal Echocardiography and Clinical Data Collection

All TOEs were performed using GE E95 equipped with Probe 6VT-D. The echocardiographic studies were reviewed and interpreted by an experienced echocardiographer blinded to the clinical data. Any oscillating CIED lead adherent structures were defined as CIED lead masses (Supplementary Videos 1–3). Demographic, clinical, and device-related data of the patients were obtained from the clinical records and retrospectively analysed.

The study was conducted according to the guidelines of the Declaration of Helsinki and was approved by the ethics committee of Brandenburg Medical School (E-02-20210621).

### Statistical Analysis

A statistical analysis was performed with IBM SPSS Statistics version 28.0 (IBM Corp., Armonk, NY). Values were expressed as mean ± standard deviation for continuous variables or as percentage for categorical variables. The variables were checked for normal distribution by the Shapiro-Wilk test and with P-P plots. The equality of variances was checked by Levene’s test of homogeneity. Significant differences between the two groups were determined by an independent samples *t*-test for continuous variables. A Pearson’s chi square test, Fisher’s exact test and a likelihood ratio were applied to detect the significant differences between the categorical variables.

## Results

In total, 1,361 TOE examinations were performed within the study period. Of these, 141 patients with CIEDs were included in the final analysis. Incidental non-infectious CIED lead masses were detected in 39 (27.6%) of the patients. The clinical characteristics of patients with and without incidental masses on CIED leads are summarised in Table 1. There were no group differences in age, sex, or cardiac risk factors, such as presence of arterial hypertension, diabetes, renal dysfunction, or CHA2DS2-VASc scores. The indications for TOE did not differ in patients with incidental CIED lead mass compared to those without incidental CIED lead mass (Table 1).

**TABLE 1:** Clinical characteristics of the study population.

	Total population *n* = 141	Incidental CIED lead mass *n* = 39	No incidental CIED lead mass *n* = 102	*P-value*
**Demographics and comorbidities**				
Age in years	73.7 ± 11.8	72.2 ± 12.4	74.4 ± 11.6	0.16
Male	91 (64.5%)	26 (66.7%)	65 (63.7%)	0.74
LVEF	39 ± 15%	33 ± 15%	41 ± 15%	0.01
Arterial hypertension	103 (73%)	26 (68.4%)	77 (75.5%)	0.39
Atrial fibrillation	115 (81.6%)	29 (74.4%)	86 (84.3%)	0.17
Diabetes type II	39 (27.7%)	11 (28.9%)	28 (27.5%)	0.86
CHA2DS2-VASc Score	3.64 ± 1.66	3.42 ± 1.64	3.72 ± 1.66	0.17
**CHA2DS2-VASc score**				
0 1 2 3 4 5 6 7	3 (2.1%) 13 (9.2%) 23 (16.3%) 20 (14.2%) 32 (22.7%) 32 (22.7%) 10 (7.1%) 5 (3.5%)	1 (2.6%) 3 (7.9%) 9 (23.7%) 7 (18.4%) 6 (15.8%) 9 (23.7%) 2 (5.3%) 1 (2.6%)	2 (2.0%) 10 (10.0%) 14 (14.0%) 13 (13.0%) 26 (26.0%) 23 (23.0%) 8 (8.0%) 4 (4.0%)	0.79
Therapeutic anticoagulation	126 (89.4%)	31 (81.6%)	95 (93.1%)	0.048
**Indication TOE**				
Assessment of VHD	88 (62.4%)	21 (53.8%)	67 (65.7%)	0.26
Exclusion of LAA-Thrombus	47 (33.4%)	17 (43.6%)	30 (29.4%)	
Exclusion of cardiac source of embolism	6 (4.3%)	1 (2.6%)	5 (4.9%)	
**Laboratory values at admission**				
NT-proBNP (pg/ml)	5116.7 ± 6524.8	5307.6 ± 5566.3	5044.9 ± 6875.9	0.42
GFR (ml/min/1.73 m^2^)	61.8 ± 27.9	65 ± 28.5%	60.49 ± 27.24	0.19
Leucocytosis (> 10,000/μ l)	10 (7.1%)	3 (7.7%)	7 (6.9%)	0.87
Increased CRP (> 5 mg/l)	52 (36.9%)	14 (35.9%)	38 (37.3%)	0.88

*CIED, cardiac implantable electronic device; NOAC, novel oral anticoagulant; LVEF, left ventricular ejection fraction; GFR, glomerular filtration rate; CRP, C-reactive protein; TOE, transoesophageal echocardiography; VHD, valvular heart disease; LAA, left atrial appendage. Mean value ± standard deviation (SD) is reported for continuous variables. Absolute number and percentage of group total are reported for the categorical variables.*

**FIGURE 2:**
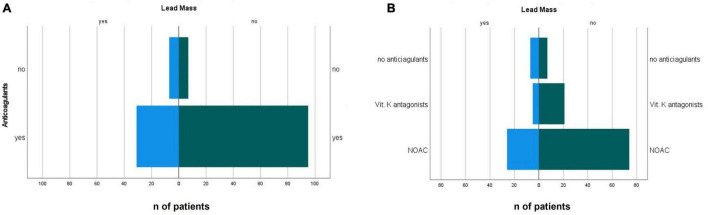
Distribution of the presence **(A)** presence of anticoagulation and **(B)** type of anticoagulant in patients with incidental non-infectious CIED lead masses and in patients with non-suspicious leads. NOAC, novel oral anticoagulant.

Patients with incidental oscillating masses on their CIED leads had a lower ejection fraction than patients without masses (33 ± 15 vs. 41 ± 15%, *p* = 0.01) (Table 1). Less patients with identified CIED lead masses were on oral anticoagulation [odds ratio 0.33, (95% CI.003–1.003), *p* = 0.048, Figure 2A]. The distribution of specified anticoagulants in patients with and without incident non-infectious CIED lead masses is shown in Figure 2B.

**FIGURE 3:**
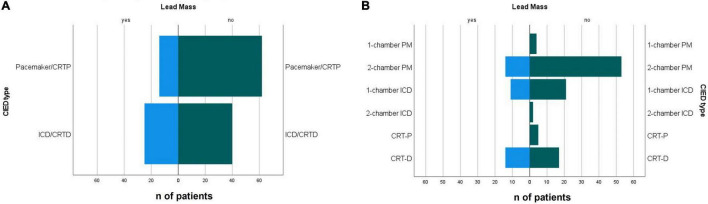
**(A)** Presence of defibrillation lead and **(B)** CIED type in patients with with incidental non-infectious CIED lead masses and non-suspicious leads. CIED, cardiac implantable electronic device; PM, pacemaker; ICD, implantable cardioverter defibrillator; CRTP, cardiac resynchronisation therapy device -pacemaker; CRTD, cardiac resynchronisation therapy device–defibrillator.

The types of CIED types indications for implantation are summarised in Table 2. Incidental CIED lead mass was more often identified on ICD and CRT-D leads compared to pacemaker and CRT-P leads [odds ratio 2.77 (95% CI 1.29–5.95), *p* = 0.008] (Table 3 and Figure 3).

**TABLE 2:** Cardiac implantable electronic device (CIED) characteristics and initial CIED indications.

	Total population *n* = 141	Incidental CIED lead mass *n* = 39	No incidental CIED lead mass *n* = 102	*P*-value
**CIED type**				
1C PM 2C PM 1C ICD 2C ICD CRT-P CRT-D	4 (2.8%) 67 (47.5%) 32 (22.7%) 2 (1.4%) 5 (3.5%) 31 (22.0%)	0 (0%) 14 (35.9%) 11 (28.2%) 0 (0%) 0 (0%) 14 (35.9%)	4 (3.9%) 53 (52.0%) 21 (20.6%) 2 (2.0%) 5 (4.9%) 17 (16.7%)	0.017
Years from implantation	7.13 ± 6.09	7.13 ± 5.28	7.13 ± 7.45	0.5
**Diagnosis at CIED implantation**				
HRCD DCM ICD	81 (57.4%) 32 (22.7%) 28 (19.9%)	14 (35.9%) 15 (38.5%) 10 (25.6%)	67 (65.7%) 17 (16.7%) 18 (17.6%)	0.004
**Indication for CIED implantation**				
Bradycardia pacing Primary SCD prophylaxis Secondary SCD prophylaxis Resynchronisation therapy	79 (56.0%) 37 (26.2%) 14 (9.9%) 11 (7.8%)	14 (35.9%) 16 (41.0%) 2 (5.1%) 7 (17.9%)	65 (63.7%) 21 (20.6%) 12 (11.8%) 4 (3.9%)	0.001

*CIED, cardiac implantable electronic device; 1C PM, one-chamber pacemaker; 2C PM, two-chamber pacemaker; 1C ICD, one-chamber implantable cardioverter defibrillator; 2C ICD, two-chamber implantable cardioverter defibrillator; CRTP, cardiac resynchronisation therapy device-pacemaker; CRTD, cardiac resynchronisation therapy device-defibrillator; HRCD, heart rhythm conduction disturbance, DCM, dilated cardiopathy; ICM, ischaemic cardiopathy; SCD, sudden cardiac death. Mean value ± standard deviation (SD) is reported for continuous variables. Absolute number and percentage of group total is reported for the categorical variables.*

**TABLE 3:** Calculated odd risks for incidental non-infectious CIED lead masses.

	OR	95% CI	*P-value*
**Demographics and comorbidities**			
Male vs. female	0.878	0.403–1.913	0.74
Presence of AF	0.54	0.22–1.32	0.17
Presence of AH	0.7	0.31–1.59	0.39
Presence of DM2	1.08	0.47–2.46	0.86
Therapeutic anticoagulation	0.33	0.11–1.003	0.048
**Diagnosis at implantation**			
DCM vs. HRCD	4.22	1.71–10.4	0.02
ICM vs. HRCD	2.66	1.01–6.97	0.047
ICM vs. DCM	0.63	0.223–1.78	0.38
**CIED type and indication**			
ICD/CRT-D vs. Pacemaker/CRT-P	2.77	1.29–5.95	0.008
Primary prophylaxis vs. bradycardia pacing	3.54	1.48–8.44	0.04
Secondary prophylaxis vs. bradycardia pacing	0.77	0.16–3.85	0.75
Resynchronisation therapy vs. bradycardia pacing	8.13	2.09–31.58	0.02
Secondary vs. primary prophylaxis	0.22	0.04–1.19	0.068
Resynchronisation therapy vs. primary prophylaxis	2.29	0.57–9.22	0.24
Resynchronisation therapy vs. secondary prophylaxis	10.5	1.51–72.1	0.033

*AF, atrial fibrillation; AH, arterial hypertension; DM2, diabetes mellitus type 2; ICD, implantable cardioverter defibrillator; CRTP, cardiac resynchronisation therapy device- pacemaker; CRTD, cardiac resynchronisation therapy device– defibrillator; HRCD, heart rhythm conduction disturbance; DCM, dilated cardiopathy; ICM, ischaemic cardiopathy.*

If further specified, 45.2% of patients with CRT-D, 34.4% with one chamber ICD, and 20.9% with two-chamber pacemaker had incidental non-infectious masses on intracardiac leads (Tables 2, 3 and Figure 3). The lifespan of the CIEDs from the first implantation to the index TOE did not differ between the groups.

**FIGURE 4:**
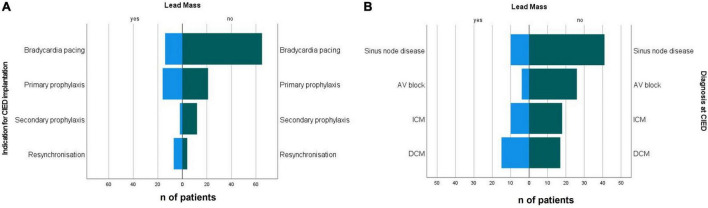
**(A)** Indications for CIED implantation and **(B)** diagnosis at the time of implantation in patients with incidental non-infectious CIED lead masses and patients with non-suspicious leads. CIED, cardiac implantable electronic device; DCM, dilated cardiopathy; ICM, ischaemic cardiopathy.

Incidental CIED lead masses were more prevalent in patients who received their device for primary prophylaxis of sudden cardiac death (43.2%) and for resynchronisation (63.6%) (Figure 4A). The prevalence of incidental CIED lead masses in patients who received their CIED as secondary prophylaxis was 14.3% and for bradycardia pacing was 17.7%. The corresponding odds ratios are provided in Table 3.

Diagnoses at CIED implantation differed between both groups, with higher prevalence of dilated cardiomyopathy (38.5 vs. 16.7%) and ischaemic cardiopathy (25.6 vs. 17.6%) (Table 2) in the group with incidental CIED lead mass. The most prevalent disease in the group with non-suspicious CIED leads was heart rhythm conduction disturbances (65.7%) (Figure 4B and Tables 2, 3).

## Discussion

In this study, we report the prevalence of incidentally detected non-infectious CIED lead masses and identify predisposing factors in a cohort of consecutive patients. According to our data, currently, non-infectious CIED lead masses are incidentally detected in 27.6% TOEs performed for indications other than suspected infectious endocarditis. This is strikingly higher, than the prevalence of 5–10% reported a decade ago (7, 8). Presumably, it could be the immensely increased quality of echocardiographic images due to introduction of higher frequency transducers and improvements in image post-processing and other technological achievements that result in such increase in the prevalence of incidental echocardiographic findings. Besides that, the number of implanted CIEDs is reported to increase steadily (1–3, 9), with concomitantly growing alertness for possible CIED-related endocarditis.

In fact, next to the feared bacterial vegetations and non-infectious adhesions to chordae tendineae and tricuspid valve, chronic fibrotic encapsulation of most long-term pacemaker, ICD, and CRT leads has been reported to develop at myocardial insertion sites, focally in the right atrium or ventricle and even along the entire length of the lead (10–15). The encapsulation sheath is composed of collagen-rich connective tissues (14, 16, 17) with endothelisation of the outer layers (13). Hence, in case the sheath is not smooth, the increasing imaging capabilities can enable us to detect it.

We presume that the higher prevalence of non-infectious incidentally detected CIED lead masses in patients with single-chamber ICD devices, when compared to pacemakers, could be due to the large uninsulated metallic surface of high-voltage defibrillation coils required to create an electrical field allowing safe defibrillation. The encapsulation sheets surrounding ICD ventricular leads have been reported to be the thickest and more often covered with fibrin thrombi and granulation (16).

An example of such encapsulation imaged by TOE can be appreciated in Supplementary Video 3. Of note, in this study, because of the retrospective analysis of “real-life” TOE loops, we could not always correctly specify the exact localisation of an adherent mass in case of multiple CIED leads.

Subsequently, in our study, the patients with incident non-infectious CIED lead masses had lower left ventricular ejection fraction and suffered more frequently from dilated cardiomyopathy or ischaemic heart disease than from heart rhythm conduction disturbances. We can presume that the combination of LV dilation and reduced systolic function resulting in altered intracardiac blood flow dynamics as demonstrated in DCM (18) might promote blood stasis in the right heart and veins, which could consequently contribute to the development of non-infectious lead encapsulation and lead masses. As defibrillation leads are mainly present in patients with reduced LVEF, it could be another explanation why oscillating masses are more prevalent on these leads.

The higher prevalence of incident CIED lead masses in patients with CRTD and ICD devices might also be one of the explanations for the higher total incidence of CIED lead masses seen in our study, as in the last decade the most notable positive trend was observed for implantations of ICDs and CRTDs (3). On the other hand, the proportions of patients with ICD/CRTD and pacemaker/CRTP in our study were comparable to the proportions reported by Downey et al. (8).

We did not observe any differences in lifespan from CIED first implantation to index TOE between the patients with and without incident lead masses in our study. This finding is in agreement with previous post-mortem findings demonstrating no relationship between extent of fibrotic encapsulation and duration since lead implantation (10). Fibrotic attachments were reported on right atrial sites of pacemaker leads as soon as 18 months after implantation (11).

The absence of therapeutic anticoagulation, especially in the presence of atrial fibrillation, should intuitively be playing a role in the formation of CIED lead masses as well. Indeed, in our study, there was a trend toward less anticoagulation in patients with non-infectious CIED lead masses. In fact, it has been proposed, using a canine model, that chronic lead encapsulation is initiated by thrombus secondary to endothelial damage and/or blood flow perturbations (17). Unfortunately, because of the low number of patients receiving vitamin K antagonists, we could not perform a statistical analysis to answer the question if these agents could be superior to novel anticoagulants in preventing the formation CIED lead encapsulations and masses.

On the other hand, the prognostic relevance of non-infectious CIED lead masses is not known. We can only speculate that if dependent on size or location, they are related to higher risks of embolism or are more prone to bacterial adhesion and, thus, development of endocarditis in the presence of systemic infection. Given the substantial risk of bleeding carried by anticoagulants, reduced LVEF and the presence of CIED leads do not present an indication for anticoagulation. Interestingly, because of the tremendous improvement in heart failure management, patients with systolic heart failure that is not caused by coronary artery disease do not seem to benefit from primary prophylactic ICD implantation in terms of reduced long-term mortality (19).

We did not observe any association between cardiovascular comorbidities, such as arterial hypertension, diabetes mellitus or chronic kidney disease, and non-infectious CIED lead masses in TOE. However, our data might be underpowered to find these differences. Some histological studies suggest that there might be an association between presence of diabetes mellitus and lead thrombosis, or between chronic kidney disease and more pronounced calcification of leads that encapsulate fibrous tissues (15). On the other hand, the echocardiographic findings, even though presumably related, are not the direct surrogate of the histological findings.

Identifying echocardiographic features that would discriminate infectious from non-infectious CIED lead masses was not the scope of our study. It has been previously reported that patients with lead vegetations were more often diagnosed with endocarditis than those with lead strands (8). However, the consequences of misdiagnosis range from missed endocarditis to unnecessary lead extraction in patients with non-infectious incidental masses on intracardiac leads. Therefore, a full clinical diagnostic workup, including inspection of CIED pocket, collection of blood cultures, checking for minor Duke criteria, and performing PET-CT in most suspicious cases (4, 20), should be started once an incidental CIED lead mass has been detected in TOE. Finally, after the endocarditis due to infectious device has been excluded, the finding of non-infectious CIED lead mass should be clearly documented for follow-ups to avoid unnecessary repeated diagnostics or even overtreatment in certain clinical situations, such as fever of unknown origin, in the future.

## Limitations

Because of national trends, the number of patients with CRT-P was low in our study. Consequently, no statement can be made if they are less prone to incident CIED lead masses than CRT-D.

Indeed, there might be an existing interaction between the low LVEF in patients with DCM and the presence of defibrillation leads in these patients, which were both related to higher prevalence of CIED lead masses. Unfortunately, a multivariate analysis was not possible because of low number of study patients. Of note, the lack of additional coagulation and RV-related parameters may have also affected the interpretability of the results. We did not include TOEs performed on patients with suspected infectious endocarditis in our study. As such, a direct comparison of echocardiographic features that would help to discriminate infectious from non-infectious CIED lead masses was not possible and remains to be answered in the future. Finally, collection of data to detect the presence of possible subclinical embolism in patients with non-infectious CIED lead masses was not possible because of the retrospective nature of this study.

## Conclusion

Incidental non-infectious CIED lead masses were frequently found in TOE, with highest prevalence in ICD and CRT-D devices implanted for patients with dilated cardiomyopathy. Patients with therapeutic anticoagulation had significantly lower prevalence of CIED lead masses than those without.

## Data Availability Statement

The raw data supporting the conclusions of this article will be made available by the authors, without undue reservation.

## Ethics Statement

The studies involving human participants were reviewed and approved by the Ethics Committee of Brandenburg Medical School. Written informed consent for participation was not required for this study in accordance with the national legislation and the institutional requirements.

## Author Contributions

TK and RJ: echocardiographic data acquisition, set up of the study, clinical data selection, statistical analysis, and manuscript preparation. CBü and JG: clinical data selection. AH-F: set up of the study, statistical analysis, and critical reading of the manuscript. MN and CBu: set up of the study and critical reading of the manuscript. All authors contributed to the article and approved the submitted version.

## Conflict of Interest

The authors declare that the research was conducted in the absence of any commercial or financial relationships that could be construed as a potential conflict of interest.

## Publisher’s Note

All claims expressed in this article are solely those of the authors and do not necessarily represent those of their affiliated organizations, or those of the publisher, the editors and the reviewers. Any product that may be evaluated in this article, or claim that may be made by its manufacturer, is not guaranteed or endorsed by the publisher.

## Supplementary Material

Supplementary Video 1 Multiplane depiction of an incidental non-infectious mass on cardiac implantable electronic device (CIED).

Supplementary Video 2 Three-dimensional echocardiographic depiction of an incidental non-infectious mass on CIED.

Supplementary Video 3 Encapsulation of an implantable cardioverter defibrillator (ICD) lead.
